# A retrospective analysis of policy development on compliance with World Health Organization’s physical activity recommendations between 2002 and 2005 in European Union adults: closing the gap between research and policy

**DOI:** 10.1186/s12889-018-5986-4

**Published:** 2018-08-31

**Authors:** X. Mayo, F. del Villar, E. Iglesias-Soler, G. Liguori, S. Mann, A. Jimenez

**Affiliations:** 1 Observatory of Healthy & Active Living of Spain Active Foundation, Centre for Sport Studies, King Juan Carlos University, Madrid, Spain; 2 Performance and Health Group, Department of Physical Education and Sport, Faculty of Sports Sciences and Physical Education, University of A Coruna, A Coruña, Spain; 3 University of Rhode Island, Kingston, RI USA; 4 Places for People, Camberley, UK; 5 Centre for Innovative Research Across the Life Cycle, Faculty of Health and Life Sciences, Coventry University, Coventry, UK; 6 GO fit LAB, Ingesport, Madrid, Spain

**Keywords:** Physical inactivity, National guidelines, Policy documents, Policy delivery

## Abstract

**Background:**

Physical inactivity (PIA) is a mortality risk factor defined as performing lower levels of physical activity than recommended by the World Health Organization (WHO). After 2002, the WHO released the WHA55.23 Resolution and the Global Strategy which produced several changes in policymaking, but with no subsequent analyses of the impact of these changes in European Union (EU) policymaking while examining PIA prevalence.

**Methods:**

PIA of 31,946 adults as a whole sample and country-by-country were analyzed in the 2002 and 2005 EU Special Eurobarometers. PIA prevalence between countries was performed with the χ2 test and PIA between both years and between genders was analyzed with the *Z*-Score test for two population proportions. A retrospective analysis of national plans was performed to interpret the suitability of such policy documents, considering changes in PIA prevalence.

**Results:**

Differences in PIA prevalence were observed between countries (*p* <  0.001) and years (*p* <  0.001) for the whole sample and men and women separately. Within-country samples showed no differences for Denmark, Finland, Ireland, Italy, Luxemburg, Portugal, and Spain (*p* > 0.05). When considering gender, there were no gender reductions in subsamples for Denmark, Finland, Ireland, Portugal, Spain, and United Kingdom, neither in Luxemburg for men, nor in France and Italy for women. When analyzing gender differences across the entire sample, PIA was higher in women than men for both years (*p* <  0.001). Greece and Luxemburg did not release national plans for promoting physical activity.

**Conclusions:**

While large differences in PIA prevalence between EU countries prevailed, the overall PIA descended between both years for the whole sample, men, and women. While this points out a general suitability of policymaking for reducing PIA, not all countries reported reductions in PIA for men, women, or both genders. Also, PIA levels were higher for women in both years, suggesting a less than optimal policy implementation, or lack of women-specific focus across the EU. This analysis helps to identify the strengths and weaknesses of PIA policymaking in the EU and provides researchers with targeted intervention areas for future development.

## Background

Physical inactivity (PIA) is a risk factor of global mortality that is defined by the World Health Organization (WHO) in terms of performing physical activity (PA) levels lower than those recommended in the *Global Recommendations* [1]. This minimum amount of PA recommended is set at a level designed to maintain an good health status, to prevent a plethora of chronic diseases, and increase life expectancy [2] and thus reduce premature death [3]. Nevertheless, examination of changes in prevalence of risk factors, particularly PIA, are rarely being analyzed concurrently with the presence and implication of national guidelines for addressing those same factors. This type of analysis is pertinent because despite the increased emphasis on reducing PIA and the importance of the evidence-based policy to inform political bodies, there is a gap between the two due to struggles to identify a policy audience [4]. In this sense, the opportunity to bring the two together is paramount for both policymakers and public health researchers and therefore close the gap between policymakers and research itself [4]. Accordingly, this body of research concurrently analyzes the compliance with PA recommendations (i.e. reduce PIA levels) and the PA policy implementation, in order to understand how national policies can help address PIA prevalence. In this sense, while countries may fulfill the policy implementation requirements requested by international bodies, it may not yield the intended results, such as reducing the prevalence of country-wide PIA [5].

After the WHO recognized in the documents the importance of the prevention and control of PIA through WHA51.17 (2000) and EB109/14 (2001), the organization urged the member states in 2002 to help developing a global strategy on PA to prevent and control noncommunicable diseases based on evidence and best practice [6]. Thus, member states were encouraged to incorporate in their national plans strategies on PA promotion [6]. The resolution ended in a request to the Director-General that would become two years later, part of a Global Strategy on Diet, Physical Activity and Health (2004) [7]. Key objectives of this global Strategy were to reduce the risk factors of PIA by means of essential public health action; to encourage the development, strengthening and implementation of both global and national policies to improve PA that were sustainable and comprehensive; and to monitor scientific data on PA to support research, including evaluation of interventions [7]. The years subsequent to 2002 should, therefore, be expected to show clear changes in policymaking and in analyzing PIA prevalence. Nevertheless, despite the European Commission completing numerous PA surveys between 2002 and 2013, there has not been any systematic analysis of change in prevalence of PIA during those years, or the implication relating these data with national PA guidelines of the member countries. At the same time, individual analysis of PIA prevalence were carried out for the years 2002 [8] and 2013 [9].

Several countries have published and developed national plans for adults that were related to PA promotion for or between 2002 and 2005, including Austria [10], Belgium [11], Denmark [12, 13], Finland [14, 15], France [16, 17], Germany [18], Ireland [19], Italy [20, 21], Netherlands [22–25], Portugal [26–28], Spain [29], Sweden [30, 31], and United Kingdom [32–34]. However, these documents had slightly different intentions and audiences, such as sustainable environment [10], public health [11–14, 17, 19–24, 26–32], sport promotion [25, 33], and active transport [15, 16, 18, 34], and not all countries place a strategic priority on PIA prevention. As a consequence, the development of documents regarding PA programs or concrete interventions to fulfill a particular action in their national plans has varied across the EU.

Thus, the primary objective of this study was to analyze the changes in PIA between 2002 and 2005 in a sample of adult individuals of the 15 member states that entered the European Union (EU) before 2004. The primary analysis was for between-country and within-country PIA levels, including rates of both men and women. The secondary objective was to relate the prevalence data to the policy implementation national guidelines of the member countries. It is anticipated that the results of our study will help to guide future changes in policy development as they relate to decreasing the prevalence of PIA across the EU, particularly in those countries newly incorporated to the EU and surveyed in 2013 for the first time.

## Methods

### Data source

In EU, the European Commission conducts public opinion surveys simultaneously on all state members of the EU to inquire about the levels of PA practice and sports participation among its citizens. These surveys were conducted in 2002, 2005, 2009, and 2013 through the *Sport and Physical Activity* and *Health and Food* Special Eurobarometers.

For the purposes of this study, data were obtained from two successive Eurobarometer surveys, December 2002 (Special Eurobarometer 183–6; *n* = 16,249) and December 2005 (Special Eurobarometer 246; *n* = 15,697), with a final sample (*n* = 31,946) from the 15 member countries that entered the EU before 2004 (Austria, Belgium, Denmark, Finland, France, Germany [combined West and East Deutschland], Greece, Ireland, Italy, Luxemburg, Netherlands, Portugal, Spain, Sweden, and United Kingdom). Due its particular characteristics, Northern Ireland was not analyzed. Besides, despite respondents in the Eurobarometers being aged 15 and over; only respondents over the age of 18 were analyzed since the PA recommendations differ between the ages [1].

Eurobarometers use a multi-stage sampling design where primary sampling units are selected from each of the administrative regions in every country. Primary sampling unit’s selection is proportional to the population size of every country from sampling frames stratified by the degree of urbanization [35].

### Measures

A modified version of the short form of the International Physical Activity Questionnaire (IPAQ) was used to determine the prevalence of PIA [36]. The IPAQ measures the intensity, frequency, and duration of the PA performed in the last 7 days. This information was obtained by the questions inquiring about the number of days practicing vigorous and moderate PA and walking activity and their respective minutes during those days. Data were analyzed following the instructions of the November’s 2005 version of the Guidelines for data processing and analysis of the IPAQ short form [36] and was carried out using a modified ad hoc spreadsheet for analyzing such data [37]. Only individuals with at least one valid intensity and duration of a particular intensity (i.e., both variables with a different answer than “don’t know”) were eligible for further analysis.

Briefly, assuming that vigorous and moderate intensity and walking represent 8.0, 4.0, and 3.3 metabolic equivalents [36], individuals were considered physically active individuals when performing (a) at least 3 days of vigorous intensity activity of at least 20 min per day, (b) at least 5 days of moderate intensity activities and/or walking for at least 30 min per day, or (c) at least 5 days combining the aforementioned intensities achieving at least 600 MET-minutes/week. Individuals not reaching any of those thresholds were considered to have a low PA level, thus being classified as physically inactive.

### Retrospective analysis of policy development

For the sake of the study, we understood policy development as the delivery of formal and informal rules and standards that defined priorities for action, goals, and strategies, as well as accountabilities of involved actors and allocation of resources that has the objective to tackle PIA [38, 39]. This policy development might have carried out in on the field of competence or another by national governments or government agencies. In this sense, we focus on written rules and standards, acquiring the usual range of acts or policy documents generally referred to as national plans [40]. For the retrospective analysis, previous literature analyzing the EU countries reporting national plans were examined in order to interpret the scope and suitability of such policy documents [40–42]. As a result, we focused on sustainable environment, public health, sport promotion, and active transport fields of competence and how they might have affected PA promotion. We analyzed 22 key variables in the policy documents of each country in order to identify the suitability of the overall national policy based on indicators of a review published elsewhere [38]. Variables analyzed and reported included (a) the use of an international normative framing regarding recommendations, indicators of (b) monitoring of PIA, (c) frame and structure of the policy, and (d) efficient coordination between bodies, (e) the use of alternatives strategies to tackling PIA prevalence, and (f) targeting concrete groups that are particularly inactive.

The initial analysis was the use of an international normative framing regarding recommendations (i.e., naming the WHA53.17, EB109/14, WHA55.23 [6], or the Global Strategy [7] of the WHO while publishing a document).

Next, we analyzed certain variables regarding monitoring of PIA were collected, such as (a) a quantitative goal (i.e., measurable) for reducing the prevalence of PIA, (b) a quantitative individual recommendation based on a national or an international body, and (c) any surveillance reference linked to a national survey for tracking changes in PIA prevalence.

Further analysis included several indicators of frame and structure of the policy of a country defined by (a) a clear time frame, (b) a sentence pointing out that tackling PIA is a strategic priority area for the country, (c) a clear budget, funding, or cost estimation of the implementation of the policy, (d) a policy identity for the plan or a project or program defined by a logo or phrase, (e) the definition of particular programs or interventions to fulfill a concrete action in the document, and (f) a means of evaluation or monitory the policy progress or completion.

We also analyzed information regarding efficient coordination between bodies in the policy, such as (a) a consultation process with key stakeholders; (b) working at different levels (e.g., with regional and local governments) on the developing of the national policy, (c) creating partnership with the private sector; (d) working in a cross-departmental fashion with other ministries, secretariats, and agencies; and (e) setting clear leadership or accountability in the implementation process.

Alternative strategies to prevent PIA prevalence were analyzed in the documents published, regarding (a) the use of population literacy or dissemination of knowledge within the people about the role of PA; (b) the use of exercise referral, prescription, or advice about PA by a general practitioner; and (c) the use of active transport (i.e., walking and cycling).

And finally, the purpose of the documents for targeting particular groups was also analyzed such as in the cases of (a) elderly, (b) people with disabilities, (c) women, and (d) inactive people.

Of the 22 variables selected for analysis, we considered a binary outcome (Yes or No) if any of the policy documents of each country clearly achieved the suitability criterion for every variable.

### Statistical analysis

The prevalence of PIA individuals in European adults between countries, analyzing men and women together and separately, were analyzed with a χ2 test for 2002 and 2005. Additionally, the prevalence of PIA was analyzed between both years (2002 and 2005) for the overall EU sample and within-country (Austria, Belgium, Denmark, Finland, France, Germany, Greece, Ireland, Italy, Luxemburg, Netherlands, Portugal, Spain, Sweden, and United Kingdom), analyzing men and women together and separately, and using a *Z*-Score for two population proportions. Data are represented as a percentage (%) with the 95% confidence interval (95% CI). Two-tail, a priori alpha level was set at 0.05. Statistical analyses were performed with Microsoft Excel version 1709 (Microsoft Corporation; Redmond, Washington, United States of America).

## Results

Significant differences in the prevalence of PIA between countries for the entire 15-country sample were observed in 2002 (*n* = 16,249; χ^2^ = 292,366; DF = 14; *p* <  0.001) and 2005 (*n* = 15,697; χ^2^ = 703,692; DF = 14; *p* <  0.001). Similarly, significant differences in the prevalence of PIA between countries were also observed for men in 2002 (*n* = 7512; χ^2^ = 89,539; DF = 14; *p* <  0.001) and 2005 (*n* = 7122; χ^2^ = 219,917; DF = 14; *p* <  0.001) and women in 2002 (*n* = 8737; χ^2^ = 223,803; DF = 14; *p* <  0.001) and 2005 (*n* = 8575; χ^2^ = 523,124; DF = 14; *p* <  0.001).

When comparing PIA prevalence between 2002 and 2005 (Table 1), it can be identified that PIA was reduced between years for the overall EU sample. However, not all countries experienced reductions in PIA prevalence between those years, specifically Denmark, Finland, Ireland, Italy, Luxemburg, Portugal, and Spain.

**Table 1:** Prevalence (%) of physical inactivity adults in the European Union countries between 2002 and 2005 and countries releasing nationals plans for physical activity promotion for or between those years

	2002		2005		*Z*-score	*p*-value
	Mean	95% CI	Mean (%)	95% CI		
European Union (*n* = 31,946)	35.5%	34.8–36.2%	29.8%	29.1–30.5%	10.84	< 0.001
Country						
Austria (*n* = 1944)	41.4%	38.3–44.5%	22.3%	19.7–24.9%	9.05	< 0.001
Belgium (*n* = 2026)	45.1%	42.1–48.1%	32.9%	29.9–35.9%	5.59	< 0.001
Denmark (*n* = 1996)	25.6%	22.9–28.3%	22.9%	20.3–25.5%	1.4	0.16
Finland (*n* = 1959)	26.6%	23.8–29.4%	26.6%	23.8–29.3%	0.02	0.99
France (*n* = 1994)	45.1%	42.1–48.2%	40.3%	37.2–43.3%	2.2	0.03
Germany (*n* = 3480)	30.2%	28.2–32.2%	16.7%	14.8–18.6%	9.19	< 0.001
Greece (*n* = 1938)	33.6%	30.7–36.6%	19.9%	17.4–22.4%	6.84	< 0.001
Ireland (*n* = 1919)	36.2%	33.2–39.3%	40.1%	37–43.2%	1.75	0.08
Italy (*n* = 1963)	41.3%	38.2–44.4%	40.3%	37.2–43.4%	0.46	0.65
Luxemburg (*n* = 1043)	31.2%	27.4–35%	29.2%	25.1–33.3%	0.68	0.5
Netherlands (*n* = 1986)	24.3%	21.6–26.9%	10.8%	8.8–12.7%	7.91	< 0.001
Portugal (*n* = 1917)	39.2%	36.1–42.3%	41.1%	38–44.2%	0.86	0.39
Spain (*n* = 3810)	38.4%	35.2–41.5%	39.2%	36.1–42.3%	0.39	0.7
Sweden (*n* = 2006)	35.2%	32.2–38.2%	28.6%	25.8–31.4%	3.19	0.001
United Kingdom (*n* = 1965)	39.2%	36.1–42.2%	34.4%	31.4–37.4%	2.18	0.03

*CI* Confidence intervals

When men and women were analyzed separately (Table 2), PIA prevalence was also reduced between 2002 and 2005. However, similar to the overall EU sample, not all countries experienced reductions in PIA by gender, particularly Denmark, Finland, Ireland, Luxemburg, Portugal, Spain; and United Kingdom for men and Denmark, Finland, France, Ireland, Italy, Portugal, Spain, and United Kingdom for women.

**Table 2:** Prevalence (%) of physical inactivity in the European Union adults between men and women for 2002 and 2005 and differences in the prevalence for both genders between the same years

	Gender (sample)	2002				2005				2002–2005	
		Mean	95% CI	Z-score	*p*-value	Mean (%)	95% CI	Z-score	*p*-value	Z-score	*p*-value
European Union	Men (*n* = 14,634)	32.9%	31.9–34.0%	6.35	< 0.001	26.8%	25.8–27.8%	7.45	< 0.001	8.06	< 0.001
	Women (*n* = 17,312)	37.7%	36.7–38.7%			32.3%	31.3–33.3%			7.48	< 0.001
Country-by-country											
Austria	Men *(n* = 859)	38.7%	33.9–43.6%	1.37	0.17	17.2%	13.8–20.6%	3.76	< 0.001	7.09	< 0.001
	Women (*n* = 1085)	43.2%	39.2–47.2%			27.2%	23.3–31.1%			5.49	< 0.001
Belgium	Men (*n* = 978)	38.9%	34.7–43.1	3.87	< 0.001	30.3%	26.1–34.4%	1.70	0.09	2.83	0.005
	Women (*n* = 1048)	50.7%	46.6–54.9%			35.4%	31.2–39.6%			4.99	< 0.001
Denmark	Men (*n* = 1012)	27.2%	23.3–31.1%	1.13	0.26	22.6%	19.0% - 26.1	0.28	0.78	1.71	0.09
	Women (*n* = 984)	24.0%	20.3–27.8%			23.3%	19.5–27.1%			0.28	0.78
Finland	Men (*n* = 813)	26.1%	21.9–30.3%	1.19	0.23	29.8%	25.3–34.3%	1.02	0.31	1.19	0.24
	Women (*n* = 1146)	27.0%	23.3–30.7%			24.4%	20.9–27.8%			1.02	0.31
France	Men (*n* = 922)	42.1%	37.6–46.5%	1.87	0.06	33.8%	29.4–38.2%	3.76	< 0.001	2.58	0.001
	Women (*n* = 1072)	47.9%	43.7–52.2%			45.6%	41.4–49.8%			0.77	0.44
Germany	Men (*n* = 1612)	30.2%	27.2–33.1%	0.02	0.92	17.4%	14.6–20.3%	0.71	0.48	5.84	< 0.001
	Women (*n* = 1868)	30.2%	27.4–33.0%			16.1%	13.5–18.6%			7.11	< 0.001
Greece	Men (*n* = 892)	30.6%	26.4–34.7%	2.02	0.04	16.5%	13.0–20.1%	2.28	0.02	4.9	< 0.001
	Women (*n* = 1046)	36.7%	32.4–41.0%			22.4%	19.0–25.9%			5.08	< 0.001
Ireland	Men (*n* = 885)	32.0%	27.8–36.3%	2.61	0.009	35.4%	30.9–40.0%	2.63	0.008	1.08	0.28
	Women (*n* = 1034)	40.2%	35.8–44.5%			43.8%	39.6–48.0%			1.19	0.24
Italy	Men (*n* = 851)	37.2%	32.9–41.5%	2.54	0.01	29.5%	24.8–34.1%	5.4	< 0.001	2.37	0.02
	Women (*n* = 1112)	45.1%	40.8–49.4%			47.0%	43.0–51.0%			0.62	0.53
Luxemburg	Men (*n* = 461)	25.1%	19.9–30.3%	2.94	0.003	29.9%	23.5–36.3%	0.26	0.79	1.15	0.25
	Women (*n* = 582)	36.5%	31.1–41.9%			28.8%	23.5–34.1%			1.99	0.05
Netherlands	Men (*n* = 978)	23.7%	19.9–27.4%	0.44	0.66	13.0%	10–16%	2.25	0.02	4.31	< 0.001
	Women (*n* = 1008)	24.9%	21.1–28.6%			8.6%	6.1–11%			6.91	< 0.001
Portugal	Men (*n* = 819)	36.8%	32.3–41.4%	1.38	0.17	37.2%	32.3–41.4%	2.02	0.04	0.12	0.9
	Women (*n* = 1098)	41.2%	37.0–45.4%			43.7%	37.0–45.4%			0.84	0.4
Spain	Men (*n* = 1712)	34.8%	30.4–39.2%	2.14	0.03	35.7%	31.1–40.3%	1.93	0.052	0.27	0.79
	Women (*n* = 2084)	41.6%	37.3–46.0%			41.8%	37.7–45.9%			0.06	0.95
Sweden	Men (*n* = 1027)	33.0%	28.7–37.3%	1.4	0.16	27.2%	30.9–23.5%	1.12	0.26	2.02	0.04
	Women (*n* = 979)	37.2%	33.1–41.4%			30.3%	26.1–34.6%			2.27	0.02
United Kingdom	Men (*n* = 799)	36.6%	31.5–41.8%	1.16	0.24	30.3%	26.1–34.4%	2.61	0.009	1.89	0.06
	Women (*n* = 1166)	40.5%	36.7–44.2%			38.2%	34.0–42.4%			0.79	0.42

*CI* Confidence intervals

When analyzing gender differences (Table 2), PIA prevalence in the overall EU sample was higher in women compared to men in both 2002 and 2005. Higher levels of PIA for women varied by country, and by year. In 2002, women in Belgium, Greece, Ireland, Italy, Luxemburg, and Spain had higher PIA prevalence than men. In 2005, women had higher PIA levels in Austria, France, Greece, Ireland, Italy, Netherland, Portugal, and United Kingdom when compared with men.

Analysis considering the key indicators of content about the national plans related to PA promotion for or between 2002 and 2005 years are developed in Table 3.

**Table 3:** Retrospective analysis of the policy documents of each country for or between 2002 and 2005 regarding the suitability of several variables regarding (a) the use of an international normative framing regarding recommendations, (b) several indicators of monitoring physical inactivity, (c) frame and structure of the documents, and (d) efficient coordination between bodies, (e) the use of alternatives strategies to tackling physical inactivity, and (f) targeting concrete groups that are particularly inactive

	International normative framing	Indicators of monitoring			Indicators of frame and structure						Indicators of efficient coordination					Alternative strategies			Target groups			
		Quantitative reduction in prevalence	Quantitative Individual recommendations	Surveillance	Time frame	Strategic priority	Economical cost of the implementation	Identity	Programs or interventions within	Evaluation of the policy	Consultation	Different levels	Private sector	Cross-departmental	Leadership and accountability	Literacy and dissemination	Exercise referral, prescription or advice by General Practitioners	Active transport	Elderly	People with disabilities	Women	Inactive people
Austria [10]	No	No	No	No	No	No	No	No	No	No	No	No	No	No	No	No	No	No	Yes	No	No	No
Belgium [11]	Yes	No	Yes	No	Yes	Yes	No	Yes	No	No	No	Yes	Yes	No	No	Yes	No	No	No	No	No	No
Denmark [12, 13]	No	No	Yes	Yes	Yes	No	No	No	No	Yes	No	Yes	Yes	Yes	Yes	Yes	Yes	No	Yes	No	No	No
Finland [14, 15]	No	Yes	No	No	No	No	Yes	No	Yes	No	Yes	Yes	Yes	Yes	Yes	Yes	Yes	Yes	Yes	Yes	No	No
France [16, 17]	Yes	Yes	Yes	No	Yes	No	No	No	No	No	No	No	No	Yes	No	No	No	Yes	No	No	No	No
Germany [18]	No	No	Yes	No	Yes	Yes	Yes	Yes	No	Yes	Yes	Yes	Yes	Yes	Yes	No	No	Yes	Yes	No	No	No
Greece	No national policies were published for or between 2002 and 2005.																					
Ireland [19]	No	No	No	Yes	Yes	No	Yes	No	No	Yes	No	No	No	Yes	No	No	No	No	No	No	No	No
Italy [20, 21]	No	No	No	No	No	No	No	No	No	No	No	No	No	No	No	No	No	No	No	No	No	No
Luxemburg	No national policies were published for or between 2002 and 2005.																					
Netherlands [22–25]	Yes	Yes	Yes	Yes	Yes	Yes	Yes	No	Yes	Yes	Yes	Yes	Yes	Yes	Yes	No	No	No	Yes	Yes	No	Yes
Portugal [26–28]	No	Yes	No	Yes	Yes	Yes	No	No	Yes	Yes	No	No	Yes	Yes	No	No	Yes	No	Yes	No	No	No
Spain [29]	Yes	No	Yes	No	No	No	No	No	No	Yes	Yes	Yes	Yes	Yes	Yes	Yes	Yes	No	No	No	No	No
Sweden [30, 31]	Yes	No	Yes	Yes	No	Yes	Yes	No	Yes	Yes	Yes	Yes	Yes	Yes	Yes	Yes	Yes	Yes	Yes	Yes	Yes	Yes
United Kingdom [32–34]	No	Yes	Yes	Yes	Yes	Yes	Yes	No	Yes	Yes	Yes	Yes	Yes	Yes	Yes	Yes	Yes	Yes	Yes	No	Yes	No

Yes: At least one document of a country achieved the suitability criterion for a particular variable

No: None document of a country achieved the suitability criterion for a particular variable

## Discussion

The main findings of the present study are that: (a) there are differences in the prevalence of PIA between countries for the whole sample and when men and women are analyzed separately during both 2002 and 2005; (b), there was a reduction in PIA prevalence in the overall EU sample between 2002 and 2005 and when men and women were analyzed together and separately, although some countries did not report such reductions; and lastly, (c) gender differences were observed for the overall EU sample for both years and also within some countries, having women higher levels of PIA than men.

The reduction in the PIA prevalence in between 2002 and 2005 indicates a likely positive effect of national PA policy guidelines for PA promotion within the EU countries [40]. Nevertheless, not all the countries that had a national policy or national guidelines achieved a reduction in PIA prevalence. Some countries, such as Denmark and Finland, already had low levels of PIA, so their lack of decrease may be reflective of strong previous public health and policy efforts [12–15]. In the case of Denmark, two public health initiatives were developed [12, 13] in which they defined qualitative targets (also by groups) and strategic lines of work, plus were already evaluating PIA prevalence. Nevertheless, no specific budgets to tackle PIA were reported, and at the same time they pointed out the necessity of developing new indicators for the PA surveillance [12, 13]. In the case of Finland [14, 15], between those years a specific document was developed to promote pedestrian and bicycle traffic. Efforts were focused on active transport, as this is the most popular place for exercise in Finland [15]. While PA in leisure time was progressively increasing, walking and cycling were decreasing, so this was a country-specific strategy for improving commuting PA levels [15].

Given the already low levels of PIA, the need is for more all-round, integrated and sustained policies that will continue to keep the population active. Additionally, even though the quality of the policy documents in both countries is good, there seems to be a lack of specificity in targeting PIA levels of the most inactive individuals. Oddly, a previous study pointed out that the policy documents focusing on inactivity people, the people who actually most need the policy, are scarce [43]. This evidences the challenge of reducing PIA prevalence in industrialized countries when low levels of PIA were already achieved, despite systematic and long-term policies are executed [44].

Additionally, countries with national plans such as Ireland [19], Portugal [26–28], and Spain [29], with a large PIA prevalence, did not reduce the PIA percentage despite having a defined PA policy from a public health perspective [19, 26–29]. Ireland, for instance, removed the quantitative goal of reducing PIA prevalence in their public health policy document, despite it being clearly pointed out in the previous version (1994). Additionally, despite a general description on coordination, frame, and structure of the policy documents, the process description was vague and general, lacking alternative strategies for reducing PIA prevalence. Similarly, Portugal had three documents in that period of time [26–28] and did not set any reasonable and reachable reduction levels for PIA and surveillance methods were not precise. Besides, their documents had limitations in structure and coordination of the policy, such as a defined budget or clear leadership and accountability of the different bodies [28]. Spain’s policy clearly lacked a frame and structure of the policy development, was vague in the explanation of the process, and the plan was carried out only in the last year of the analyzed period [29].

In contrast, some countries produced reductions in the PIA prevalence with national policy documents related to PA and released during those years, such as Belgium [11], France [16, 17], United Kingdom [32–34], and notably Sweden [30, 31] and Netherlands [22–25]. Belgium’s policy treated PA with a transversal consideration in the different lines of work and focused on enabling an environment that helped PA promotion [11]. France worked in their policies with quantitative targets in mind about the reduction in PIA prevalence and individual recommendations, plus developed a cycling policy pointing out the importance of PA in health promotion [16, 17]. The case of the United Kingdom is worth noting, since this country released several documents focusing on PA promotion [32–34]. For instance, a national plan for walking and cycling (2004), with the aim of increase PA as a key public health intervention, explaining strategic lines to do so and particular budget lines to carry out those working lines [32–34]. They also developed two documents focused on sport promotion and its effect on health, with clear objectives to achieve on a time frame, a clear funding scheme, and strategic lines and policy implementation recommendations to work on in the long run. Nevertheless, the weakest part of the documents was the evaluation structure of the achievement of objectives and fulfillment of strategic lines and recommendations, despite naming it [32, 33]. Additionally, and such as the case of Finland, they also pointed out the need of standardizing data collection in relation to PA participation and attitude change indicators [33]. Further, while Sweden was able to reduce PIA prevalence of both genders while maintaining the equality in the PIA prevalence of between genders, Netherlands reduced more the PIA prevalence in women in comparison with men. In this sense, it is important to note that in both cases PA promotion was treated as a prioritized area [23, 30, 31]. In the particular case of Sweden, PA objectives (i.e., national objective 9) were just qualitative, but public health plans implementation pursued specifically gender equality in sports participation with a public health perspective [31] and in health itself [45]. Regarding Netherlands, with good policy documents, feasible objectives in PIA prevalence were clearly stated [23], some particular budgets for PA promotion were defined [24], and some target groups for PA promotion were defined [24].

On the other hand, some countries achieved reductions in the PIA prevalence despite just having partial national policy documents related with transport (i.e., cycling), such as Germany (2002), or sustainable development, such as Austria (2002). In this sense, the transport national plan of Germany is an example of well-defined policy document, stated specific goals and targets, a budget defined for the implementation of the program, and an evaluation process specified; always for the cycling promotion [18]. Additionally, it clearly stated the levels of work (i.e., national, sub-national and local) and the cross-departmental nature (i.e., different ministries and agencies) of the implementation regarding public bodies. At last, leadership and accountability of agents were very clearly defined, particular programs were stated, and the elderly population was targeted as a group [18]. Regarding Austria, the sustainable development national guidelines did not include direct references, and it lacked national guidelines for PA promotion [10]. In this regard, it seems that reductions in PA prevalence might have been more related with the start-up of the autonomous Sports Ministry between 2000 and 2003 and programs for the whole country such as *Active Aging and Strengthening*, which may have helped to reduce of the PIA prevalence between those years [10].

For countries without national PIA-reduction plans, outcomes varied. Greece saw reductions in PIA prevalence for the entire population was observed, yet in Luxemburg such reductions were not identified. The secular trends in PIA for countries without policy implementation should be understood as a maintaining of the prevalence of PIA. The same can be said for the insufficient implementation of policies, as could be identified in Italy, who just named PA in their documents [20, 21], or Portugal or Spain, each with crucial flaws in their documents. In this sense, the results observed for Greece are surprising and may be related to the government structure (regional or local governments with strong competencies in PA promotion), the population distribution, or differences in the sampling between the Eurobarometers.

Although there was overall gender-based reductions in PIA prevalence and within many of the countries, the patterns were not consistent. In some countries, there was a PIA prevalence reduction in women while reducing at the same time the prevalence of PIA in men, such as in Austria, Belgium, Germany, Greece, Netherlands, and Sweden. Nevertheless, this was not the case in other countries. France and Italy reduced the PIA prevalence in men but not in women, suggesting an insufficient involvement of women, likely due to some combination of lacking policy, access, or encouragement in the policy development focused on them. Austria did realize a reduction in PIA for both genders, however it was much greater in men. These three cases, and since no countries showed larger reductions in women, suggest a greater ability to influence men in the policy-making and policy implementation, which points out the necessity of strengthening the development of women-focused PA policy and implementation, as was previously observed [43]. Further, while some national policies mentioned gender as a variable in designing PA policies, none quantified a plausible or desirable reduction in the PIA prevalence in women. Lastly, in Luxemburg only was observed a reduction in the prevalence of PIA in women.

Several changes in policymaking were carried out in the form of national policies and guidelines as a consequence of the WHA55.23 Resolution [6] and the Global Strategy on Diet, Physical Activity and Health [7] between 2002 and 2005. Nevertheless, there were not previously published analyses on how these documents could have determined changes in PIA prevalence. Data and analysis arising from this article are therefore valuable and relevant information that can be taken into account by policymakers, helping therefore to close the gap between research and policy [4]. In this sense, to ensure that policy implementation is translated into PIA reductions, a close and consistent cooperation among stakeholders is needed, in which researchers should have themselves a role, particularly presenting valuable and implementable data and conscientious analyses. Additionally, all-round policies not just for health and sports, but also in education, transport, and urban planning need to be crafted such that gender differences are addressed. When policies that promote PA are successfully integrated, the default option for citizens should be to choose a healthy lifestyle, subsequent to this, the prevalence of PIA is reduced [46].

One limitation worth noting is that the comparisons between different Special Eurobarometers are limited due to differences in questions and methods of collecting answers. The Special Eurobarometers of 2002, 2005, and 2013 collected data through the IPAQ, but this was not the case during the Special Eurobarometer of 2009. Furthermore, while Special Eurobarometers of 2002 and 2005 used raw minutes as responses to PA questions, the Special Eurobarometer of 2013 only stratified the answers in time blocks. Thus, differences observed with the 2009 and 2013 Special Eurobarometers limits the comparability for just between 2002 and 2005. Previously, individuals analysis of PA prevalence in Special Eurobarometers was carried out for the years 2002 [8] and 2013 [9], but neither analyses between years were performed nor those data were related with the implication at the level of national guidelines of those country members. Additionally, our study is limited in the sense that there might exist a latency between the publication of WHO documents, the implementation of the policies, and the changes in the prevalence of PIA, as can be observed in the fact that only five countries out of 15 used a WHO normative framing regarding PIA while publishing a document. Considering that possible comparisons are limited between 2002 and 2005 due to methodological differences, more long-term changes in assuming the WHO normative framing and prevalence of PIA cannot be analyzed.

## Conclusions

Large differences in PIA prevalence existed between EU countries for years 2002 and 2005, but the overall prevalence of PIA was reduced between both years. Nevertheless, when data were analyzed country-by-country, some countries did not report reduced PIA and some showed more humble reductions for women compared to men, which could indicate a less than optimal policy development and implementation in some countries. Some noted policy limitations include not indicate quantitative goals in individual and prevalence terms, not treating PIA as a priority area, not clearly indicating available funding or means to assess the policy intervention, not using alternatives ways for promoting positive behavior or not targeting particular groups such as women or inactive population per se. These analyses in PIA prevalence may be an interesting tool for analyzing the strengths and weaknesses of the policymaking and development reported in each respective PA promotion policy national plans between those years and how those impacted in the current prevalence of PIA of their citizens.

Taking all this into account, the analyses of changes in the prevalence of PIA and the role that national plans and guidelines may have is of crucial importance because they allow the review of the suitability of PA policies of each European country and the EU as a whole. This information can then be used to inform Health Secretariats about the estimated health expenditure and disease prevalence in each respective country due to the burden of PIA [47]. As a consequence of this, complying with the voluntary reductions of PIA within the *Global action plan for the prevention and control of noncommunicable diseases* should be of primary importance for each state member [48].

## Abbreviations

CI: Confidence interval
IPAQ: International Physical Activity Questionnaire
PA: Physical activity
PIA: Physical Inactivity
WHO: World Health Organization
